# RETRACTED: Di Paola et al. Assessment of 2-Pentadecyl-2-oxazoline Role on Lipopolysaccharide-Induced Inflammation on Early Stage Development of Zebrafish (*Danio rerio*). *Life* 2022, *12*, 128

**DOI:** 10.3390/life14091115

**Published:** 2024-09-05

**Authors:** Davide Di Paola, Sabrina Natale, Enrico Gugliandolo, Marika Cordaro, Rosalia Crupi, Rosalba Siracusa, Ramona D’Amico, Roberta Fusco, Daniela Impellizzeri, Salvatore Cuzzocrea, Nunziacarla Spanò, Fabio Marino, Alessio Filippo Peritore

**Affiliations:** 1 Department of Chemical, Biological, Pharmaceutical and Environmental Science, University of Messina, 98166 Messina, Italy; davide.dipaola@unime.it (D.D.P.); sabrina.natale@unime.it (S.N.); rsiracusa@unime.it (R.S.); rdamico@unime.it (R.D.); rfusco@unime.it (R.F.); dimpellizzeri@unime.it (D.I.); fabio.marino@unime.it (F.M.); aperitore@unime.it (A.F.P.); 2 Department of Veterinary Science, University of Messina, 98166 Messina, Italy; egugliandolo@unime.it (E.G.); rcrupi@unime.it (R.C.); 3 Department of Biomedical and Dental Sciences and Morphofunctional Imaging, University of Messina, 98166 Messina, Italy; cordarom@unime.it; 4 Department of Pharmacological and Physiological Science, School of Medicine, Saint Louis University, Saint Louis, MO 63103, USA

The journal retracts the article, “Assessment of 2-Pentadecyl-2-oxazoline Role on Lipopolysaccharide-Induced Inflammation on Early Stage Development of Zebrafish (*Danio rerio*)” [1], cited above. 

Following publication, concerns were brought to the attention of the Editorial Office regarding duplicated figures across this article [1] and two earlier publications [2,3] from a similar authorship group.

Adhering to our standard procedure, an investigation was conducted that confirmed duplications between panels contained in Figure 1A in paper [1] and Figure 1 within [2], and Figures 2 and 3 in the published paper [3]. While the authors cooperated with the Editorial Office during the investigation, they were unable to satisfactorily explain the overlap of the figures, nor could they provide the original raw material for Editorial Board evaluation. As a result, the Editorial Board has lost confidence in the reliability of the findings and decided to retract this publication [1], and as per MDPI’s retraction policy (https://www.mdpi.com/ethics#_bookmark30).

This retraction was approved by the Editor-in-Chief of *Life*.

The authors disagree with this retraction.
